# Imidazolium-Functionalized Ionic Porous Organic Polymer for Efficient Removal of Oxo-Anions Pollutants from Water

**DOI:** 10.3390/molecules30030473

**Published:** 2025-01-22

**Authors:** Wei Huang, Hong Zhong, Junyue Lin, Xiaodan Li, Jie Mao, Hongliang Dai, Yuntong Li, Shengchang Xiang

**Affiliations:** 1 College of Chemistry and Materials Science, Fujian Normal University, Fuzhou 350007, China; huangwei@jgsu.edu.cn; 2 Key Laboratory of Jiangxi Province for Special Optoelectronic Artificial Crystal Materials, School of Chemistry and Chemical Engineering, Jinggangshan University, Ji’an 343009, China; zhonghbush@jgsu.edu.cn (H.Z.); 9919960006@jgsu.edu.cn (J.L.); lixiaodan@jgsu.edu.cn (X.L.); daihongliang@jgsu.edu.cn (H.D.); 3 School of Environment and Energy Engineering, Anhui Jianzhu University, Hefei 230000, China; maojie@ahjzu.edu.cn

**Keywords:** ionic porous organic polymers, adsorption, oxo-anions pollutants, selectivity

## Abstract

The development of highly efficacious materials for the removal of toxic heavy metal-based oxo-anions is of utmost importance. Herein, an ionic porous organic polymer (designated as HB-IPOP) was synthesized through a quaternization reaction between hexa(imidazole-1-yl)benzene and 5,5′-Bis(bromomethyl)-2,2′-bipyridine. HB-IPOP exhibited high saturation uptake capacities, specifically 292 mg·g^−1^ for Cr_2_O_7_^2−^ and 531 mg·g^−1^ for ReO_4_^−^, and demonstrated exceptional selectivity for both Cr_2_O_7_^2−^ and ReO_4_^−^. Additionally, HB-IPOP demonstrates high recyclability, allowing its reuse over at least five cycles. DFT calculations confirmed that the superior interaction sites and binding energies of HB-IPOP with Cr_2_O_7_^2−^ and ReO_4_^−^ outperform the affinities of other competing anions. This theoretical validation aligns with the experimentally observed high capacity and selectivity of HB-IPOP for these oxo-anions. Hence, HB-IPOP emerges as a promising candidate to replace current adsorbent materials in the effective removal of Cr_2_O_7_^2−^ and TcO_4_^−^ anions from water.

## 1. Introduction

Water pollution, an unfortunate consequence of relentless industrialization and technological advancement, poses a formidable obstacle to human health and ecological sustainability, prompting urgent attention to purify contaminated water resources [1,2]. In the assessment of the U.S. Environmental Protection Agency, oxo-anions dominate as the primary pollutants in water. Notably, the International Agency for Research on Cancer recognizes hexavalent chromium [Cr (VI)] and its oxoanionic form as Group 1 carcinogens, sparking widespread concern due to their toxicity, bioaccumulation potential, and long-term health implications [3,4,5,6,7]. In addition, the proliferation of radioactive byproducts from nuclear power plants also poses a formidable environmental challenge. Among these byproducts, technetium-99 (^99^Tc), primarily present as pertechnetate ions (TcO_4_^−^), has garnered significant attention due to its long half-life [8,9,10]. The daunting task of mitigating environmental contamination caused by TcO_4_^−^ stems from the formidable challenges posed by its exceptional water solubility and remarkable mobility, making it a persistent and pervasive threat to aquatic ecosystems and groundwater resources [11,12]. In light of the formidable challenges posed by these contaminants, the urgent development and implementation of highly efficient remediation strategies are paramount.

To purify wastewater and eliminate harmful contaminants, various techniques have been developed, including adsorption, chemical precipitation, biological treatments, photocatalytic reduction, and electrodialysis [13,14,15,16,17,18,19,20]. Among these methods, adsorption excels as a promising strategy for the removal of trace pollutants due to its simplicity, safety, and unparalleled effectiveness. Porous materials like activated carbons, cellulose derivatives, zeolites, covalent organic frameworks (COFs), and metal–organic frameworks (MOFs) are widely recognized adsorbents in water remediation processes [21,22,23,24,25]. However, conventional porous materials (cellulose derivatives, activated carbons, and zeolites) frequently grapple with inadequate adsorption capacity, whereas MOFs can encounter suboptimal water stability. Additionally, the intricate preparation procedures associated with COFs present challenges that impede their widespread adoption [26]. Consequently, there arises an imperative necessity for the development of alternative adsorbents that embody both economic viability and stability, along with the crucial capability for regeneration, all while maintaining exceptional adsorption capacity and selectivity [27,28].

Porous Organic Polymers (POPs), meticulously assembled via covalent bonds, exhibit an array of advantages, including low skeletal density, exceptional thermal and chemical stability, facile and versatile synthesis routes, coupled with the capability to fine-tune and personalize their pore characteristics [29,30]. Due to their unique pore structure and chemical properties, these adsorbents exhibit great potential for removing pollutants from water. Especially when targeting specific pollutants such as oxo-anions, efficient adsorption, and separation can be achieved by designing porous organic polymers with appropriate pore size, surface chemistry, and functionality [31]. Although these compounds have garnered notable attention for their properties and potential applications in gas adsorption, catalysis, photovoltaics, and biosensors, they have limited effectiveness in removing ionic contaminants from water. As a result, scientists are increasingly focusing their endeavors on the design and fabrication of ionic porous organic polymers (IPOPs), which inherently bear a net charge on their surface within the polymeric framework [32,33,34,35,36]. The electrostatic interaction between the charged adsorbing moieties and the charged scaffold of IPOPs imparts them with a distinctive adsorptive capacity, thereby enabling their efficient removal of ionic contaminants from water. Therefore, a cationic porous organic polymer is anticipated to efficiently capture anionic contaminants in wastewater via anion exchange. Central to this process is a network of cations capable of exchanging anions, which is pivotal for sequestering anions from the solution. Studies have also shown that cationic porous organic polymers featuring quaternary ammonium nitrogen sites and exchangeable halide anions exhibit a very weak electrostatic interaction between these two components [37,38,39]. As a consequence, this unique property significantly enhances the dispersion of cationic porous organic polymers within aqueous environments, facilitating the swift and efficient sequestration of targeted anionic contaminants from water through ion exchange processes. Furthermore, imidazolium groups have strong cationic properties and can effectively bind to anionic contaminants in water through electrostatic interactions. This interaction helps to enhance the adsorption capacity and selectivity of the adsorbent for these pollutants [40,41].

**Figure 1:**
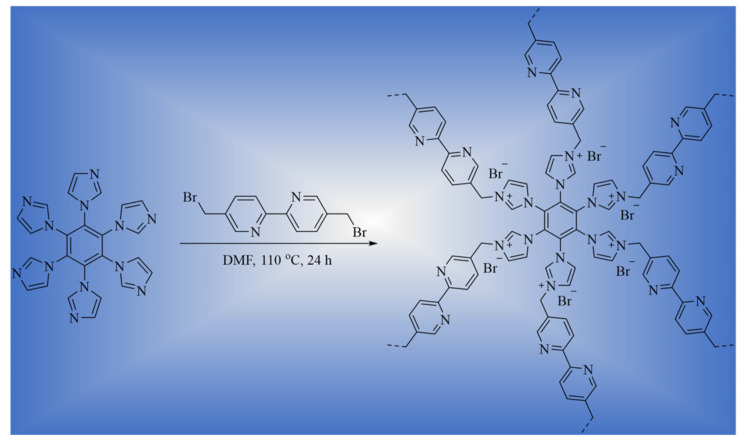
A schematic depiction of the synthesis process for HB-IPOP.

In this work, a novel cationic porous organic polymer (HB-IPOP) was fabricated via a quaternization reaction (Figure 1). Benefiting from the nitrogen-rich imidazole cores and exchangeable bromide (Br^−^) ions within its porous network structure, HB-IPOP exhibits high selectivity and effectiveness in removing Cr_2_O_7_^2−^ and ReO_4_^−^ anions from water, utilizing ReO_4_^−^ as a representative for radioactive TcO_4_^−^ in the process.

## 2. Results and Discussion

**Figure 2:**
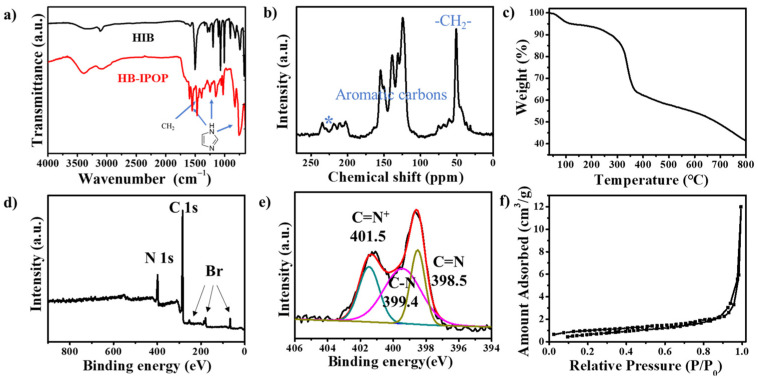
(**a**) FTIR spectra, (**b**) Solid state ^13^C NMR spectra, (**c**) TGA curve, (**d**) XPS survey spectra, (**e**) N 1s spectra, (**f**) N_2_ sorption isotherms at 77 K. * Marked peaks correspond to side bands.

The chemical compositions and structures of HB-IPOP were confirmed through Fourier-transform infrared (FTIR), solid-state ^13^C NMR spectra, and powder X-ray diffraction (PXRD). As depicted in the FTIR spectra (Figure 2a), the peaks situated at 1468, 1150, and 755 cm^−1^ are assigned to the vibrations of stretching and bending within the imidazolium ring, confirming the imidazolium units in the PILs skeleton. The peak at 1391 cm^−1^ represents the stretching frequency of the methylene (-CH_2_-) functional groups, which are essential for the growth of the ionic polymeric networks [42]. The successful synthesis of the ionic polymer is confirmed by these results. In the solid-state ^13^C NMR spectra depicted in Figure 2b, prominent peaks between 100 and 160 ppm are attributed to the aromatic carbons present within the imidazole and benzene rings. Additionally, the distinct peaks at approximately 50 ppm correspond to the methylene linkages that are bonded to the nitrogen atoms of the imidazole in HB-IPOP, which are crucial for the development and expansion of the ionic polymeric networks. TGA curves demonstrate that HB-IPOP maintains its stability up to 300 °C, while decomposition is observed after approximately 300 °C (Figure 2c). The weight loss observed below 100 °C is attributed to the desorption of guest molecules, such as water, which is expected. The contact angle indicates that HB-IPOP exhibits excellent wettability (Figure S2), ensuring effective dispersion in water for ion exchange processes (Figure S3).

The X-ray photoelectron spectroscopy (XPS) detects five clear signal categories around 69, 182, 255, 285, and 400 eV, corresponding to the Br 3d, Br 3p, Br 3s, C 1s, and N 1s levels, respectively (Figure 2d). These findings definitively establish the significant presence of free bromide ions (Br^−^) in HB-IPOP. The deconvolution peaks at 401.5, 399.4, and 398.5 eV correspond to three unique nitrogen configurations within HB-IPOP: C=N^+^, C−N, and C=N (Figure 2e). Both field-emission scanning electron microscopy (SEM) and field-emission transmission electron microscopy (TEM) reveal that HB-IPOP predominantly exhibits a granular morphology (Figures S4 and S5). Additionally, EDX spectra and elemental mapping verify the coexistence of free bromide ions (Br^−^) alongside carbon (C) and nitrogen (N) elements (Figures S6 and S7). PXRD patterns further corroborate the amorphous nature of the HB-IPOP structure, consistent with the characteristics of numerous other documented ionic polymers (Figure S8). These comprehensive datasets mutually support each other, furnishing strong evidence for the successful fabrication of the targeted polymer.

The porosities of HB-IPOP were investigated via the N_2_ adsorption–desorption tests at 77 K; HB-IPOP displays type IV adsorption–desorption isotherms (Figure 2f). A steep increase of N_2_ (P/P_0_ > 0.91) and a notable hysteresis can be observed, indicating the coexistence of mesopores and macropores. The application of nonlocal density functional theory (NLDFT) to the pore size distribution derived from the adsorption branch supports the notion that mesopores and macropores dominate the polymer structure. The pore size distribution of the resultant samples depicted in Figure S9 shows multi-peaks ranging from 2 to ~15 nm together with a wide peak centered around 80 nm. The Brunauer–Emmett–Teller (BET) surface area of HB-IPOP is 3.34 m^2^·g^−1^, with a corresponding pore volume of 0.0166 cm^3^·g^−1^; such a small value might be attributed to the inclusion of flexible linkages lacking the necessary stiffness to sustain a stable porous structure. Alternatively, the occupation of cavities by counterions could also be a factor contributing to the low surface area.

**Figure 3:**
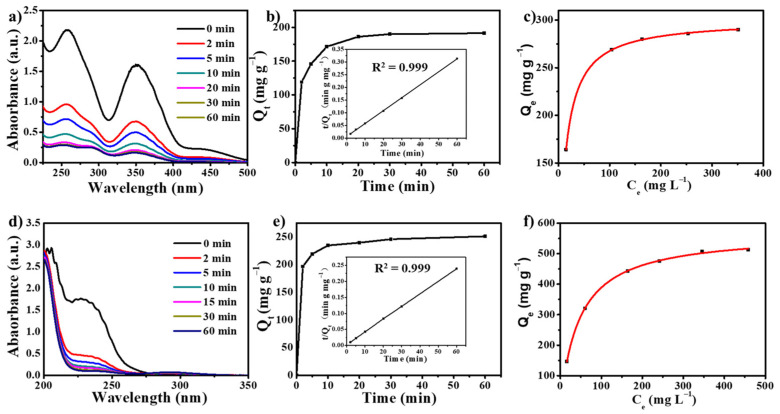
Temporal UV-Vis spectroscopy of (**a**) Cr_2_O_7_^2−^, and (**d**) ReO_4_^−^aqueous solution in the presence of HB-IPOP. Kinetic analysis of the capture of (**b**) Cr_2_O_7_^2−^ and (**e**) ReO_4_^−^. Equilibrium adsorption isotherms of (**c**) Cr_2_O_7_^2−^ and (**f**) ReO_4_^−^ onto HB-IPOP with Langmuir fits.

After examining the intrinsic structural properties of the composite polymer, we assessed its ability to adsorb anionic contaminants. The abundance of freely mobile Br^−^ ions provides a favorable setting for investigating the ion adsorption process. Moreover, the wettability and stability of the compound in conventional aqueous environments present promising avenues for exploration. Initially, we aimed to evaluate the effectiveness of HB-IPOP as an adsorbent for removing Cr (VI) oxide anions, which are mainly present as CrO_4_^2−^ and Cr_2_O_7_^2−^. To achieve this, an extensive investigation was carried out on the time-dependent removal of Cr_2_O_7_^2−^ using HB-IPOP as the adsorbent material. As depicted in Figure 3a, 10 mg of the sample was submerged in 20 mL of a 0.5 mM Cr_2_O_7_^2−^ aqueous solution, and the characteristic peak at 258 nm was tracked over time via UV-Vis spectroscopy. As observed, the UV-Vis spectra showed a decreasing trend as time elapsed, indicating the adsorption of Cr_2_O_7_^2−^ anions. The adsorption process was rapid, with approximately 57% of the Cr_2_O_7_^2−^ being removed within the initial 2 min, and nearly 87% removal was accomplished within 60 min (Equation (1)). Additionally, a visible shift from yellow to nearly colorless was observed in the solutions after 60 min, further confirming the swift capture of Cr_2_O_7_^2−^ (Figure S10a).

To assess the efficacy of HB-IPOP as an adsorbent for other anionic contaminants, we carried out additional experiments targeting the adsorption of surrogate compounds that mimic toxic and radioactive contaminants, such as TcO_4_^−^. Given the risks of radioactivity and the complexity of handling, ReO_4_^−^ was selected as a non-radioactive alternative to TcO_4_^−^ because of their similar size, charge distribution, and structure. Analogous to the detection of Cr_2_O_7_^2−^, UV-Vis spectroscopy can be employed to track the variations in the characteristic peak at 230 nm (λmax for ReO_4_^−^) over time. For ReO_4_^−^, the removal efficiency is exceptionally high, achieving approximately 74% removal within the first 2 min and nearly 94% within 60 min (Figure 3c). The swift perrhenate removal capability exhibited by HB-IPOP makes it a promising option for the elimination of TcO_4_^−^. Upon analyzing the adsorption curves, we noticed that HB-IPOP showed a faster adsorption rate for ReO_4_^−^ compared to Cr_2_O_7_^2−^, which may be due to the dual negative charges and relatively larger molecular size of Cr_2_O_7_^2−^ (Figure S11). As a result, we believe that the hierarchical pore structure of HB-IPOP is more favorable for facilitating the exchange of single negatively charged, small-molecular-volume oxygen-containing anions. As further evidence, ion exchange due to electrostatic interaction between the cation framework and anion underpins the adsorption mechanism in our material (Figure S19).

The pseudo-first-order kinetic equation (Equation (2)) (Figures S12 and S13) and the pseudo-second-order kinetic equation (Equation (3)) were the two rate equations chosen to investigate the kinetic behavior. It is noteworthy that the capture of anions by the HB-IPOP material adhered to pseudo-second-order kinetics, with the process being entirely reliant on the amounts of adsorbent and adsorbate, as evidenced by the time-dependent adsorption capacity. As illustrated in Figure 3b,e, the interactions of the HB-IPOP with both anions followed a pseudo-second-order kinetic model with a correlation coefficient (*R*^2^) above 0.999. Furthermore, the rate constant (*k*_2_) values for HB-IPOP with Cr_2_O_7_^2−^ and ReO_4_^−^ ions were calculated to be 0.00391 mg·g^−1^·min^−1^ and 0.00481 mg·g^−1^·min^−1^, respectively. Additionally, we assessed the capacity of HB-IPOP to adsorb oxo-anions using the Langmuir adsorption isotherm model. As shown in the adsorption isotherms (Figure 3c,f), the adsorption isotherms demonstrated a strong agreement between the experimental data and the Langmuir equation (Equation (4)), suggesting that the oxo-anions were adsorbed in a monolayer fashion onto the surface of the HB-IPOP network. Given the broad pore size distribution and the strong adsorptive capabilities of its cationic framework, HB-IPOP exhibited a remarkably high maximum adsorption capacity for the targeted anions (Tables S1 and S2). Specifically, the respective maximum adsorption capacities for Cr_2_O_7_^2−^ and ReO_4_^−^ were found to be 292 mg·g^−1^ and 531 mg·g^−1^.

**Figure 4:**
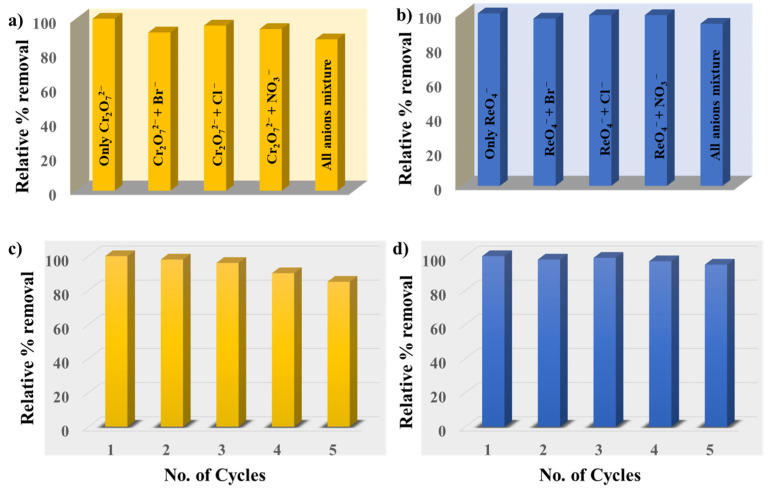
The bar diagrams illustrate the efficiency of HB-IPOP in removing (**a**) Cr_2_O_7_^2−^ and (**b**) ReO_4_^−^ in the presence of anions like Cl^−^, Br^−^, and NO_3_^−^. Recyclability test of compound for (**c**) Cr_2_O_7_^2−^, (**d**) ReO_4_^−^.

Motivated by the exceptional adsorption properties of HB-IPOP, we sought to evaluate its selectivity for targeted oxo-anions in the context of common interfering anions like Br^−^, Cl^−^, and NO_3_^−^, which are typically found in real wastewater streams. The comparative experiments were conducted using a variety of coexisting anions, each mixed in a 10:1 ratio with either Cr_2_O_7_^2−^ and ReO_4_^−^ oxo-anions separately. Despite competition from other anions, HB-IPOP exhibited remarkable selectivity for Cr_2_O_7_^2−^ and ReO_4_^−^ with their removal efficiency remaining nearly constant (approximately 100%) in each instance (Figure 4a,b). This underscores the exceptional binding capacity of HB-IPOP towards these oxo-anions. Apart from catalytic activity and selectivity, the recyclability of catalysts is equally vital in heterogeneous catalytic systems. Elution studies revealed that anionic contaminants adsorbed on HB-IPOP can be efficiently desorbed using sodium hydroxide (NaOH) elution, leading us to choose 0.5 M NaOH as the preferred eluent. Subsequently, the adsorbent was soaked in 0.5 M HBr to restore the counteranion to Br^−^, facilitating the reset of its adsorptive properties for subsequent use. As depicted in Figure 4c,d, the adsorption efficiency of the polymer remained relatively stable, with no significant changes observed over the first three cycles. However, as the reaction progressed, the capture rate declined slightly, mainly attributed to the increasing material loss of HB-IPOP as the reaction progressed over time. Additionally, during the reaction, some metal ions occupied the active sites, resulting in a decreased capture rate in subsequent cycles.

**Figure 5:**
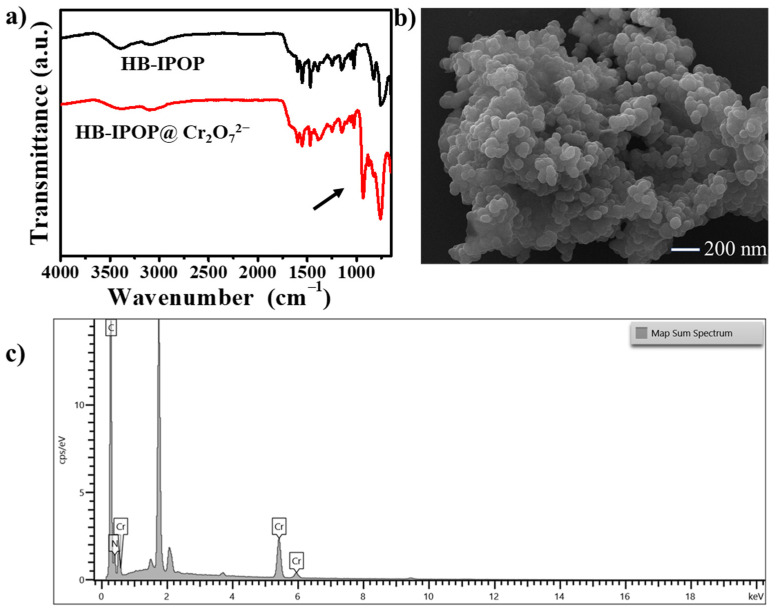
(**a**) The FTIR spectrum of HB-IPOP adsorbed Cr_2_O_7_^2−^. (**b**) SEM morphology image and (**c**) EDX elemental analysis of HB-IPOP adsorbed Cr_2_O_7_^2−^.

We conducted analyses using FTIR, SEM, EDX spectra, and elemental mapping on the polymer that had adsorbed contaminated anions to enhance our understanding of its structural stability. A side-by-side comparison was conducted between the adsorbed and original FT-IR spectra. As illustrated in Figure 5a, the ionic polymer exhibited characteristic infrared peaks at 940 cm^−1^ following the adsorption of Cr_2_O_7_^2−^ ions, and the polymer that had adsorbed ReO_4_^−^ displayed peaks at 910 cm^−1^ (Figure S14) [11,43,44]. Moreover, the retention of all other bands, corresponding to those of the original HB-IPOP, suggests that the structural integrity of HB-IPOP remains intact even after the adsorption of diverse anionic pollutants. The FE-SEM images of the adsorbed polymer closely resemble those of the original HB-IPOP sample (Figure 5b and Figure S16). This observation further confirms that the HB-IPOP framework remains essentially unaltered following the capture of various anionic pollutants. The EDX spectra of the adsorbed polymer revealed the presence of the intended contaminants (Cr, Re), which indicates that Br^−^ ions are being replaced by anions during the exchange process (Figure 5c and Figure S17). The elemental mapping analyses further demonstrated a uniform distribution of the intended contaminants, verifying their successful incorporation within the pores of HB-IPOP (Figures S15 and S18).

**Figure 6:**
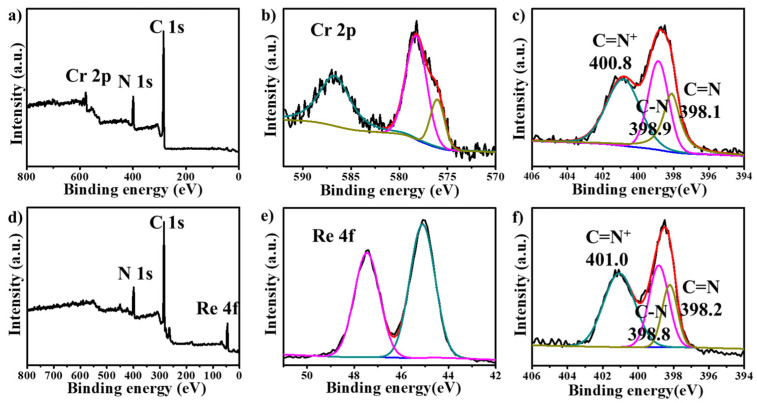
XPS survey spectra of HB-IPOP adsorbed (**a**) Cr_2_O_7_^2−^, (**d**) ReO_4_^−^. XPS spectra of (**b**) Cr 2p and (**e**) Re 4f for HB-IPOP adsorbed Cr_2_O_7_^2−^, ReO_4_^−^. N 1s for HB-IPOP-adsorbed (**c**) Cr_2_O_7_^2−^, (**f**) ReO_4_^−^.

To gain a deeper insight into the adsorption mechanism and changes in functional groups of the material, we conducted the XPS analysis on the adsorbed material. As illustrated in Figure 6a,d, the XPS analysis conclusively verifies the existence of constituent elements Cr (Figure 6b) and Re (Figure 6e) within the material upon adsorption of the respective oxygen-containing anions. By comparing the N1s core-level spectra before and after adsorption, we observed a notable shift in the N1s core-level spectra of the adsorbed material relative to HB-IPOP (Figure 6c,f). This shift suggests a strengthened electrostatic interaction between the negatively charged oxyanions and the positively charged cation framework.

**Figure 7:**
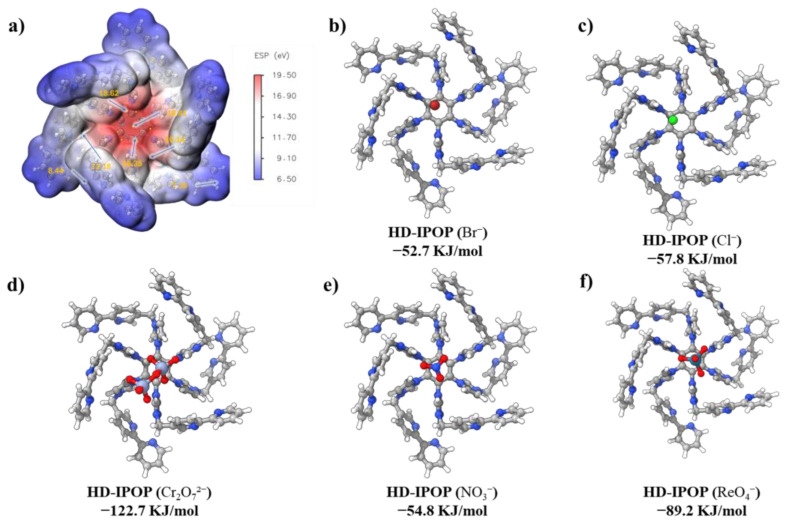
ESP maps of monomer unit of HB-IPOP (**a**). The binding energies of the monomeric unit of HB-IPOP with (**b**) Br^−^, (**c**) Cl^−^, (**d**) Cr_2_O_7_^2−^, (**e**) NO_3_^−^, (**f**) ReO_4_^−^.

To further validate and solidify our findings regarding selectivity and affinity of HB-IPOP, we employed Density Functional Theory (DFT) calculations, which exhibited a strong correlation with our experimental results. As depicted in Figure 7a, the electrostatic potential (ESP) map of the monomers demonstrates a harmonious alignment with the balanced charge distribution surrounding the benzene and imidazole rings. Notably, the imidazole ring exhibits high potential and serves as the primary binding site for oxo-anions. Furthermore, DFT calculations provided profound insights into the superior binding affinity of Cr_2_O_7_^2−^ and ReO_4_^−^ ions, compared to other anions, as demonstrated by their respective binding energy values (Figure 7b−f). As shown in Figure 7d, the binding energy of Cr_2_O_7_^2−^ with the structural units of HB-IPOP was found to be 122.7 kJ·mol^−1^, substantially higher than that of other anions. The robust interactions and high affinity of HB-IPOP for ReO_4_^−^ are evident from the calculated binding energy of 89.2 kJ·mol^−1^ between ReO_4_^−^ and the structural units of HB-IPOP. In contrast, the binding energy between Br^−^ and the structural units of HB-IPOP is 52.7 kJ·mol^−1^, significantly lower than that of the targeted analytes. This relatively low binding energy offers advantages to the material in kinetics, capture efficiency, and selectivity.

## 3. Materials and Methods

Hexa(imidazole-1-yl)benzene was prepared as described in the literature [45]. The remaining chemicals were sourced from commercial supplier Adamas-beta and were used without further purification.

### 3.1. Synthesis of HB-IPOP

HB-IPOP was synthesized via a quaternization reaction. A solution comprising hexa(imidazole-1-yl)benzene (HIB) and 5,5′-Bis(bromomethyl)-2,2′-bipyridine (BIP) in DMF was agitated at 110 °C for 24 h, and a yellow precipitate was observed. The obtained precipitate was then separated and sequentially washed with ethanol, water, acetonitrile, and dichloromethane to remove any unreacted monomers and residues to the greatest extent possible. Subsequently, the precipitate was dried under vacuum (Figure S1).

### 3.2. Characterization

The Fourier transform infrared (FT-IR) spectra were obtained using the Perkin-Elmer instrument (Waltham, MA, USA), with readings collected between 400 and 4000 cm^−1^. Solid-state ^13^C nuclear magnetic resonance (^13^C-NMR) analysis was performed on the Bruker SB Avance III 500 MHz spectrometer (Bruker, Karlsruhe, Germany). Thermogravimetric analysis (TGA) was conducted using the NETZSCH STA 449C system (Netzsch GmbH, Selb, Germany), with heating in an N_2_ airflow at a continuous rate of 10 °C per minute, ranging from room temperature (rt) to 800 °C. Nitrogen sorption isotherms were measured using the ASAP 2460 system (Micromeritics, Norcross, GA, USA). Powder X-ray diffraction (XRD) patterns of the as-prepared samples were collected with a powder X-ray diffractometer (Cu Kα radiation source, Miniflex600, Rigaku, Tokyo, Japan). Field-emission scanning electron microscopy (SEM) was carried out on the JEOL JSM-7500F instrument (JEOL, Tokyo, Japan). Transmission electron microscope (TEM) images were captured using the JEOL JEM-2010 instrument (JEOL, Tokyo, Japan). X-ray photoelectron spectroscopy (XPS) measurements were recorded on the Thermo ESCALAB 250 spectrometer (Thermo Fisher Scientific, Waltham, MA, USA).

### 3.3. Time-Dependent Analysis of Oxo-Anions Removal

In the anion exchange experiment with Cr_2_O_7_^2−^, 10 mg of HB-IPOP was dispersed in a 20 mL aqueous solution of Cr_2_O_7_^2−^ (0.5 mmol·L^−1^). The mixture was stirred at room temperature, and liquid UV-Vis spectroscopy was used to measure the Cr_2_O_7_^2−^ concentration at the typical absorption peak of 258 nm after various adsorption durations. In a comparable manner, we analyzed the UV-Vis spectra of ReO_4_^−^ ions, utilizing their respective typical absorption peaks of 230 nm for ReO_4_^−^ as reference points. Through time-dependent analysis, the rate of removal and the decline in oxo-anion concentration were calculated.

### 3.4. Calculation of Capacity

HB-IPOP (10 mg) sample was immersed in a 5 mL solution of oxo-anions (5 mM) and continuously stirred for 24 h. By utilizing the initial and final absorbance measurements, we calculated the saturation uptake capacity of HB-IPOP over 24 h using the provided equation.

(1) $$Q_{t}=\frac{\left(C_{0}-C_{t}\right)\times V}{m}$$

Specifically, C_0_ represents the initial concentration of oxo-anions, C_t_ denotes the concentration at various time points, Q_t_ indicates the adsorption capacity, V stands for the volume of the solution, and m signifies the mass of the adsorbent employed.

### 3.5. Kinetic Model

#### 3.5.1. The Pseudo-First-Order Kinetic Model

The linear form of the pseudo-first-order kinetic model is expressed by the following equation.

(2) $$ln(Q_{e}-Q_{t})=lnQ_{e}-k_{1}t$$

where Q_t_ and Q_e_ are the amount of pollutants adsorbed at time and equilibrium (mg·g^−1^), k_1_ is the pseudo-first-order rate constant of the adsorption process (min^−1^).

#### 3.5.2. The Pseudo-Second-Order Kinetic Model

The linear form of the pseudo-second-order kinetic model is expressed by the following equation.

(3) $$\frac{t}{Q_{t}}=\frac{1}{k_{2}{Q_{e}}^{2}}+\frac{t}{Q_{e}}$$

where Q_t_ and Q_e_ are the amount of pollutants adsorbed at time t and equilibrium (mg·g^−1^), k_2_ is the pseudo-second-order rate constant of the adsorption process (g·mg^−1^·min^−1^).

### 3.6. Absorption Isotherm Experiment

Separate 2 mg portions of HB-IPOP were submerged in 4 mL of aqueous solutions containing different concentrations of the respective oxo-anion (specifically, Cr_2_O_7_^2−^ ions ranging from 25 to 500 ppm and ReO_4_^−^ ions ranging from 50 to 700 ppm). The filtrate was analyzed by UV-Vis spectroscopy after being stirred for 2 h at room temperature. The interactive behaviors between the HB-IPOP and oxo-anions were analyzed by following the equation.

(4) $$Q_{e}=Q_{m}\frac{kC_{e}}{1+kC_{e}}$$

where K and Q_m_ are the Langmuir isotherm constants and maximum adsorption capacity, Ce signifies the concentration at equilibrium, while Q_e_ signifies the adsorption capacity at equilibrium.

### 3.7. Selective Adsorption Experiments

For our study on anion capture, we incorporated Br^−^, Cl^−^, and NO_3_^−^ as interfering ions, as these ions are abundant in typical water sources, especially wastewater. We immersed 2 mg of HB-IPOP in 4 mL of an aqueous Cr_2_O_7_^2−^ solution, where the concentrations of Cl^−^, Br^−^, and NO_3_^−^ ions were each 10 times higher than that of Cr_2_O_7_^2−^. The solution was stirred at room temperature for 24 h, subsequently filtered to separate the adsorbent from the aqueous solution, and then analyzed using UV-Vis spectrophotometry. The efficiency of HB-IPOP in removing oxo-anions in the presence of competing anions was assessed by comparing their concentrations to those in the original Cr_2_O_7_^2−^ solution. A similar procedure was applied to the ReO_4_^−^ solution.

### 3.8. Recyclability Test

Once the anion exchange process for oxo-anions was completed, the solid powder was isolated and subsequently released using a 0.5 M solution of NaOH. The solid powder, after release, was regenerated with 0.5 M HBr solution. The recyclability of the regenerated materials was assessed with 0.5 mM solutions containing oxo-anions (Cr_2_O_7_^2−^, ReO_4_^−^). The same process, consisting of adsorption and desorption, was carried out 5 times consecutively.

### 3.9. Computational Details

Using the ORCA 5.0.3 software package, DFT calculations were conducted. The energy calculations and geometry optimizations were carried out using the composite approach B97-3c [46]. After the IPOP fragment structure was individually optimized, the IPOP fragment was fixed in the anion-bound complex for restrictive optimization to avoid deformation of the framework structure. Electrostatic potential grid data files are generated with the Multiwfn program and visualized with the VMD program [47].

## 4. Conclusions

In summary, a cationic porous organic polymer (HB-IPOP) featuring pyridinic and imidazole rings has been successfully synthesized via the quaternization reaction for water remediation. HB-IPOP exhibits rapid adsorption kinetics, high removal efficiency, and excellent selectivity for oxo-anions. The respective maximum adsorption capacities for Cr_2_O_7_^2−^ and ReO_4_^−^ were found to be 292 mg·g^−1^ and 531 mg·g^−1^. Moreover, the recyclability of HB-IPOP positions it as a promising candidate for applications in water remediation. Furthermore, DFT calculations reveal the exceptional selectivity of HB-IPOP and provide profound insights into the potential recognition sites for oxo-anions. This research offers a new protocol for the development of high-performance adsorbent materials at the molecular level.

## Data Availability

The data presented in this study are available on request from the corresponding authors.

## Supplementary Materials

The following supporting information can be downloaded at https://www.mdpi.com/article/10.3390/molecules30030473/s1, Figure S1. The photographs of HB-IPOP; Figure S2. Water contact angle measurements for HB-IPOP; Figure S3. The dispersibility photographs of HB-IPOP in H_2_O (5.0 mg/5 mL); Figure S4. SEM images of HB-IPOP; Figure S5. TEM image of HB-IPOP; Figure S6. EDX spectra of the HB-IPOP; Figure S7. Elemental mapping for HB-IPOP; Figure S8. The PXRD data of HB-IPOP; Figure S9. Pore-size-distribution of HB-IPOP calculated from the adsorption isotherm with NLDFT method; Figure S10. Visual color change of aqueous solution of HB-IPOP adsorbed (a) Cr_2_O_7_^2−^, (b) ReO_4_^−^; Figure S11. Removal (in %) of Cr_2_O_7_^2−^ and ReO_4_^−^ with HB-IPOP at different time intervals; Figure S12. The pseudo-first-order model of Cr_2_O_7_^2−^; Figure S13. The pseudo-first-order model of ReO_4_^−^; Figure S14. The FTIR spectrum of HB-IPOP adsorbed ReO_4_^−^; Figure S15. Elemental mapping of the HB-IPOP adsorbed Cr_2_O_7_^2−^; Figure S16. SEM morphology image of HB-IPOP adsorbed ReO_4_^−^; Figure S17. EDX spectra of the HB-IPOP adsorbed ReO_4_^−^; Figure S18. Elemental mapping of the HB-IPOP adsorbed ReO_4_^−^; Figure S19. The adsorption process of HB-IPOP’s removal of oxo-anions; Table S1: Comparison of Cr_2_O_7_^2−^ adsorption capacity of HB-IPOP with other adsorbents; Table S2. Comparison of ReO_4_^−^ adsorption capacity of HB-IPOP with other adsorbents. References [11,12,40,48,49,50,51,52,53,54,55,56,57,58,59,60,61,62] are cited in the Supplementary Materials.
