# Supplementary material for: Imidazolium-Functionalized Ionic Porous Organic Polymer for Efficient Removal of Oxo-Anions Pollutants from Water
Source: Molecules. 2025 Jan 22;30(3):473. doi: 10.3390/molecules30030473 (PMC11821212; doi:10.3390/molecules30030473)
Supplement: Supplementary file 1 [file molecules-30-00473-s001.zip › molecules-3424998-supplementary.pdf]

Supporting information

Imidazolium-functionalized ionic porous organic polymers for efficient removal of oxo-anions pollutants from water

Wei Huang [1,2], Hong Zhong [2], Junyue Lin [2], Xiaodan Li [2], Jie Mao [3], Hongliang Dai [2], Yuntong Li [2,*], and Shengchang Xiang [1,*]

[1] College of Chemistry and Materials Science, Fujian Normal University, Fuzhou 350007, China
[2] Key Laboratory of Jiangxi Province for Special Optoelectronic Artificial Crystal Materials, School of Chemistry and Chemical Engineering, Jinggangshan University, Ji'an, Jiangxi 343009, China
[3] School of Environment and Energy Engineering, Anhui Jianzhu University, Hei fei, Anhui 230000, China
* Correspondence: liyuntong@jgsu.edu.cn (Y.L.); scxiang@fjnu.edu.cn (S.X.)

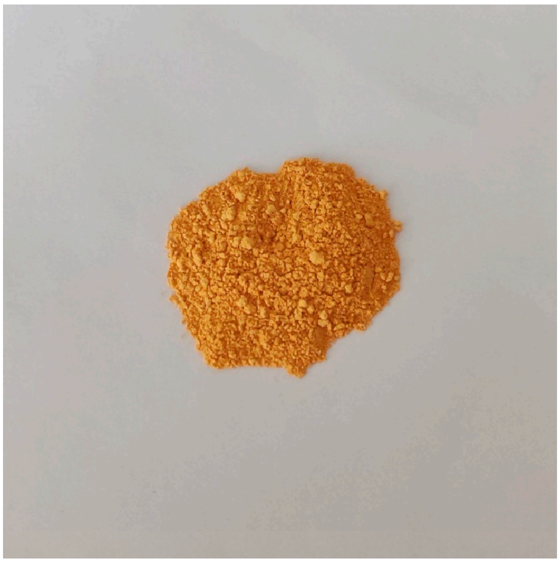

Figure S1. The photographs of HB-IPOP.

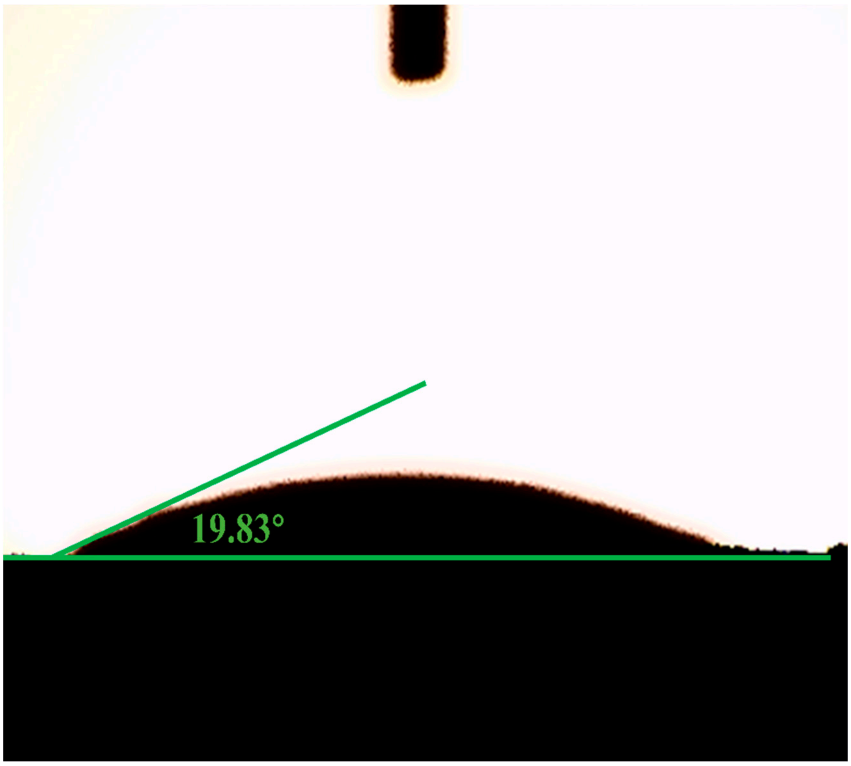

Figure S2. Water contact angle measurements for HB-IPOP.

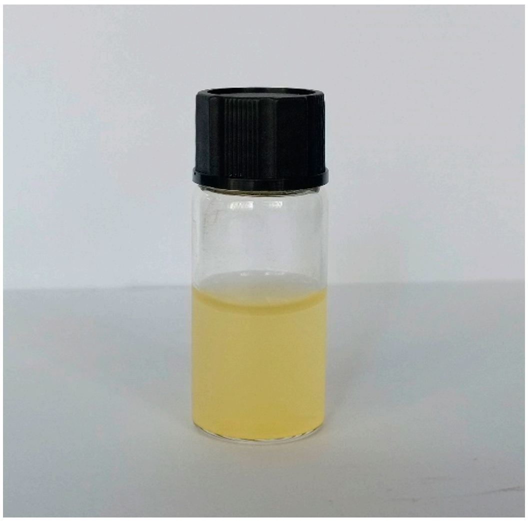

Figure S3. The dispersibility photographs of HB-IPOP in H_2O (5.0 mg/5 mL).

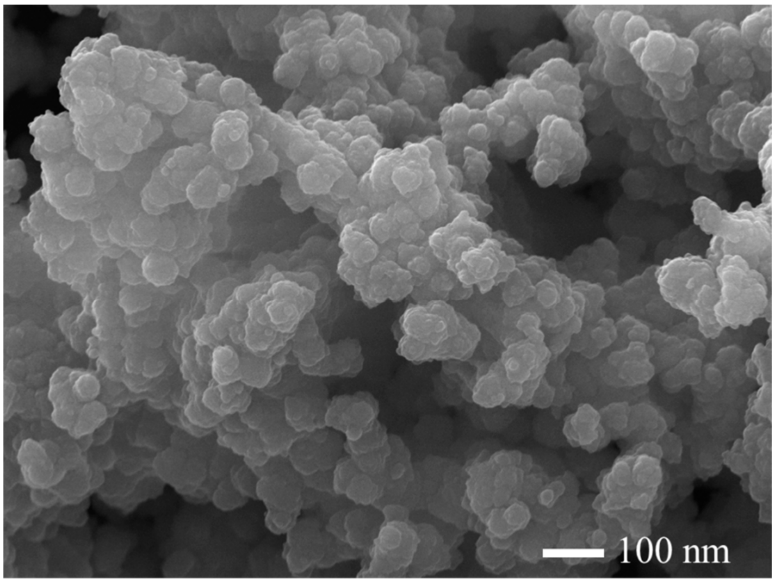

Figure S4. SEM images of HB-IPOP.

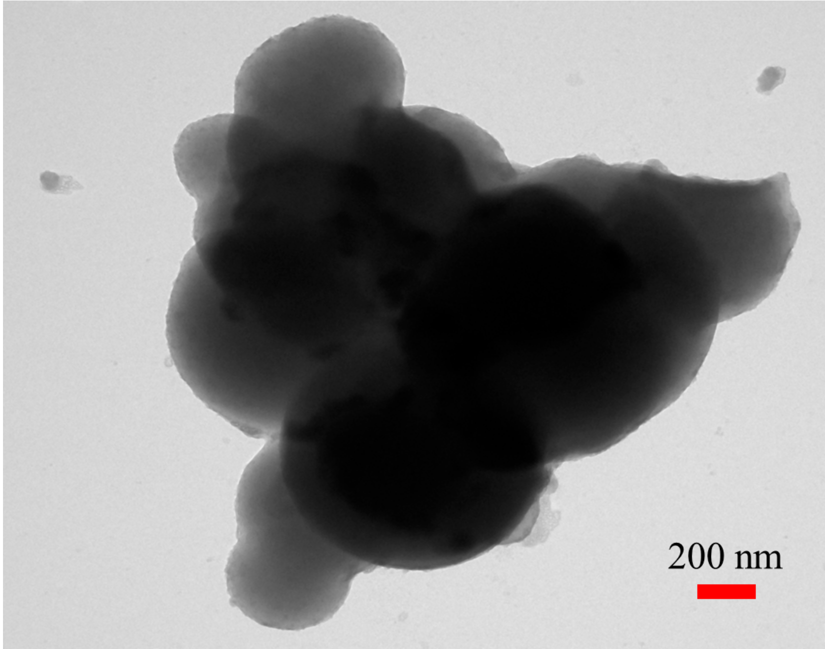

Figure S5. TEM image of HB-IPOP.

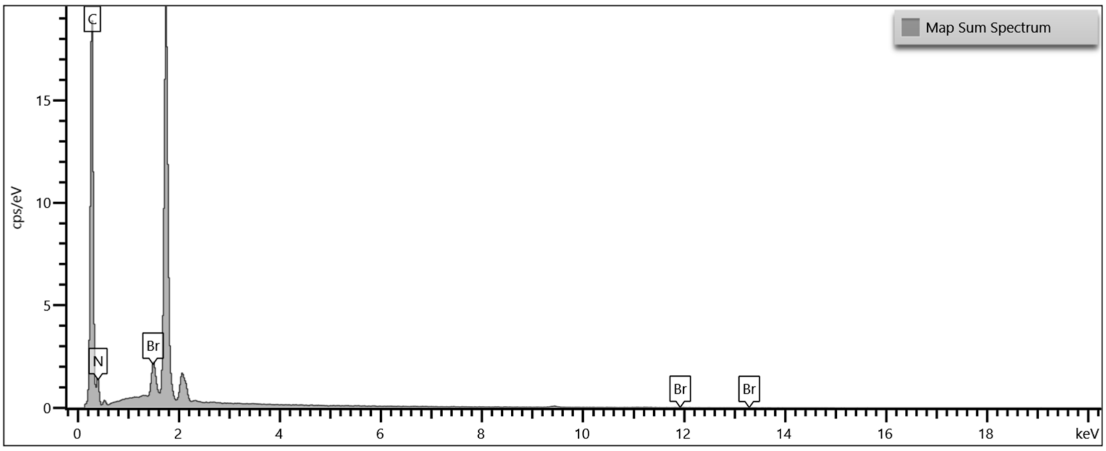

Map Sum Spectrum		
Element	Wt%	Wt% Sigma
C	70.50	0.51
N	24.47	0.54
Br	5.04	0.08
Total:	100.00	

Figure S6. EDX spectra of the HB-IPOP.

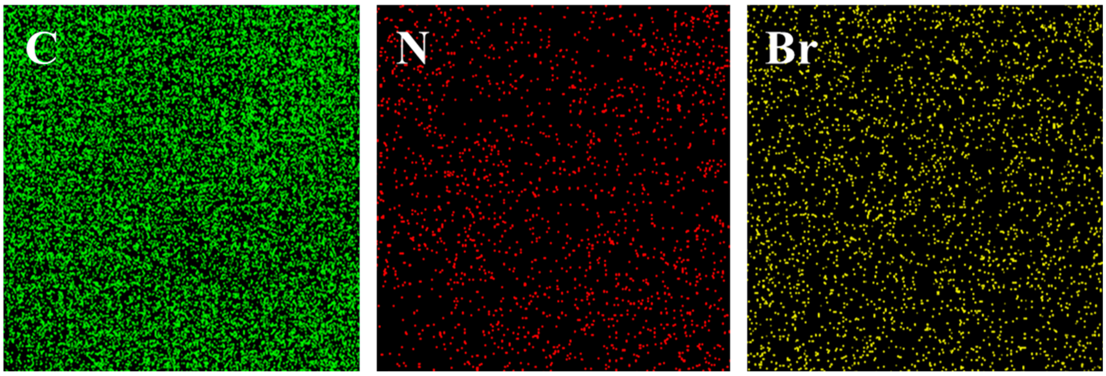

Figure S7. Elemental mapping for HB-IPOP.

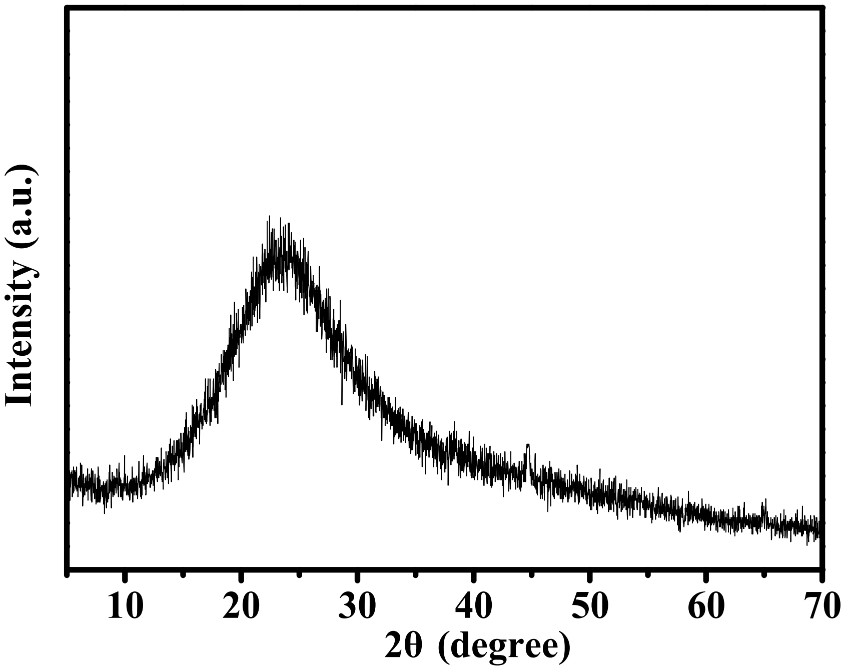

Figure S8. The PXRD data of HB-IPOP.

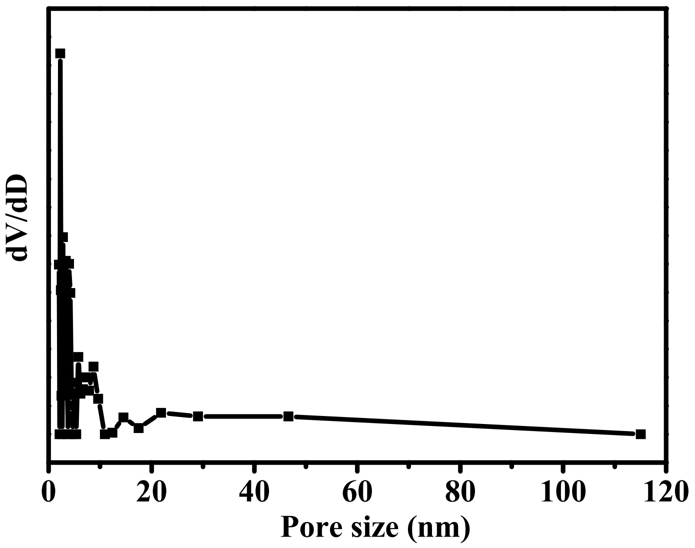

Figure S9. Pore-size-distribution of HB-IPOP calculated from the adsorption isotherm with NLDFT method.

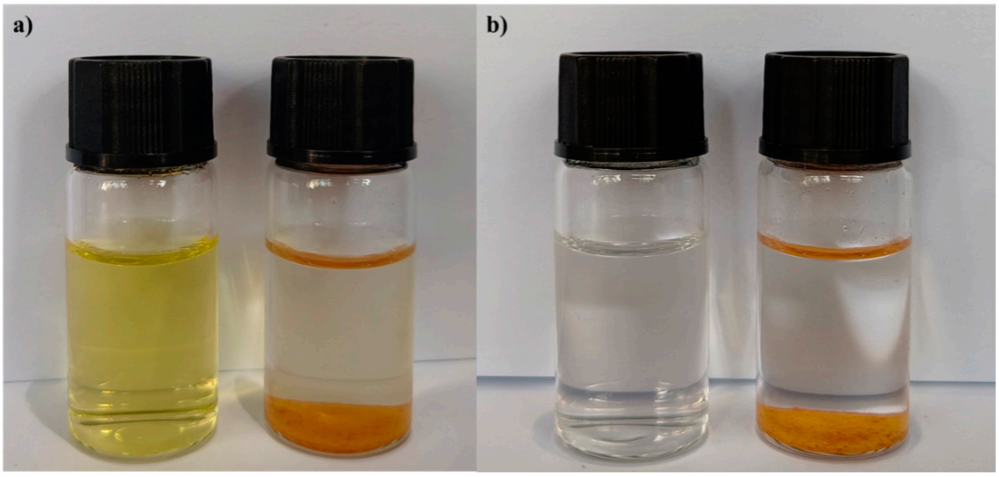

Figure S10. Visual color change of aqueous solution of HB-IPOP adsorbed (a) $Cr_2O_7^{2-}$, (b) ReO_4^-.

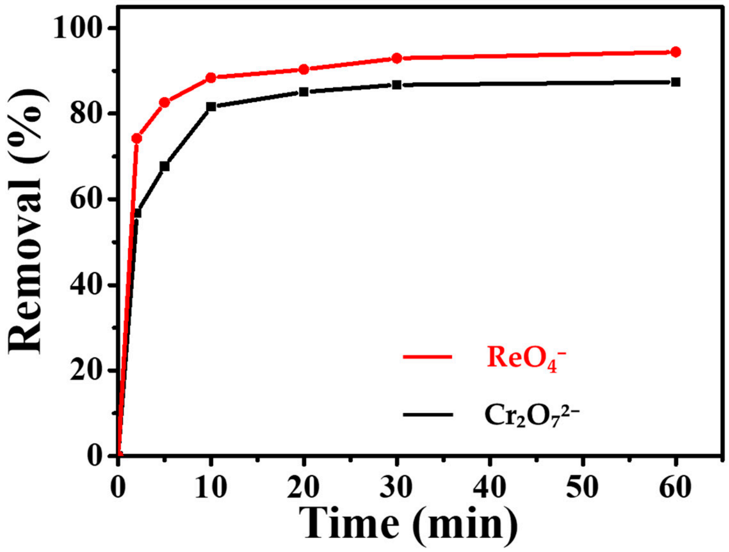

Figure S11. Removal (in %) of $Cr_2O_7^{2-}$ and ReO_4^- with HB-IPOP at different time intervals.

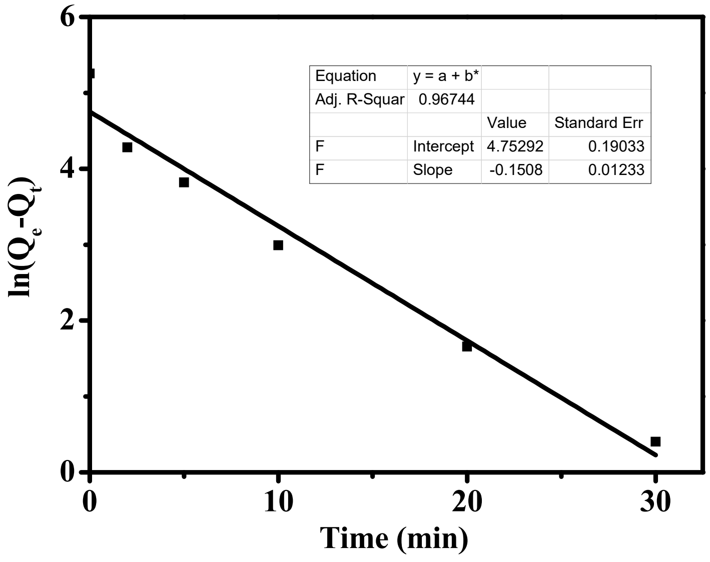

Figure S12. The pseudo-first-order model of $Cr_2O_7^{2-}$.

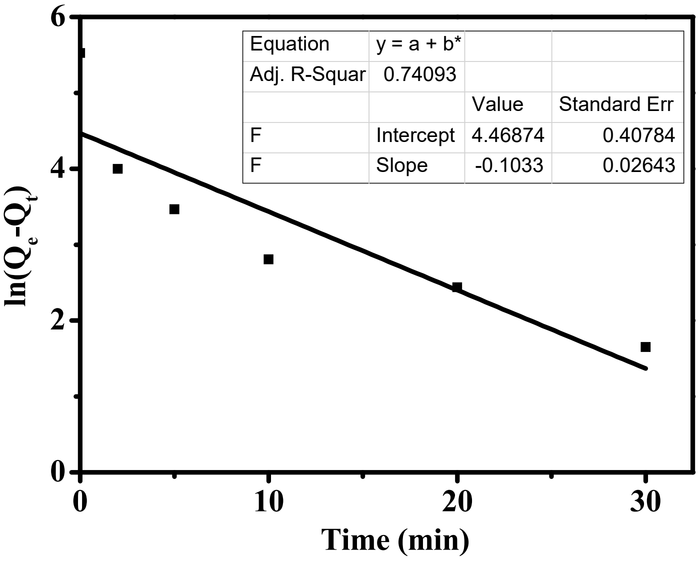

Figure S13. The pseudo-first-order model of ReO_4^-.

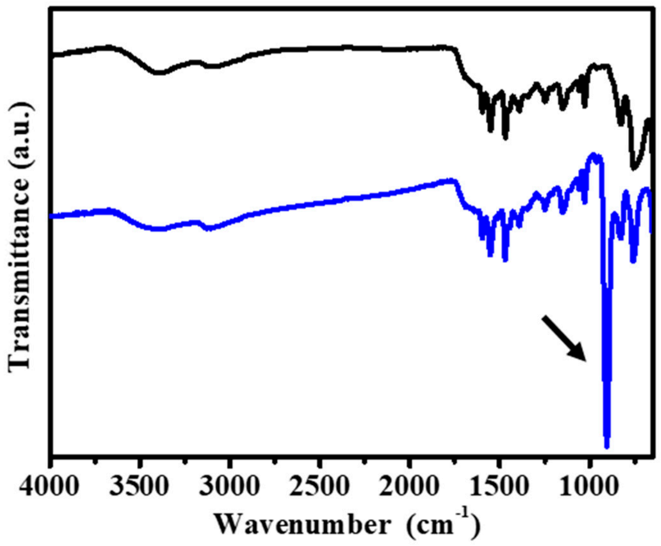

Figure S14. The FTIR spectrum of HB-IPOP adsorbed ReO_4^-.

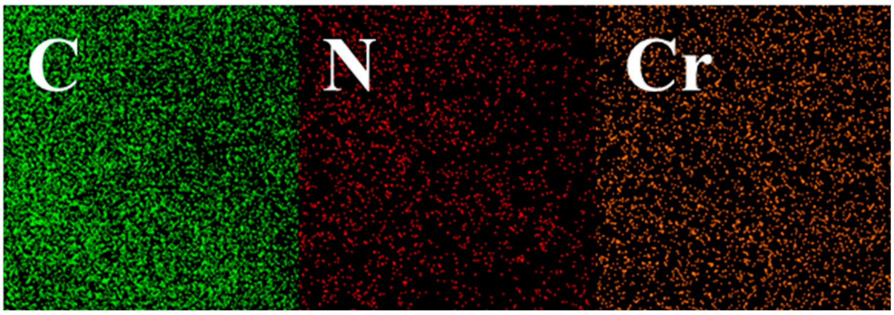

Figure S15. Elemental mapping of the HB-IPOP adsorbed $Cr_2O_7^{2-}$.

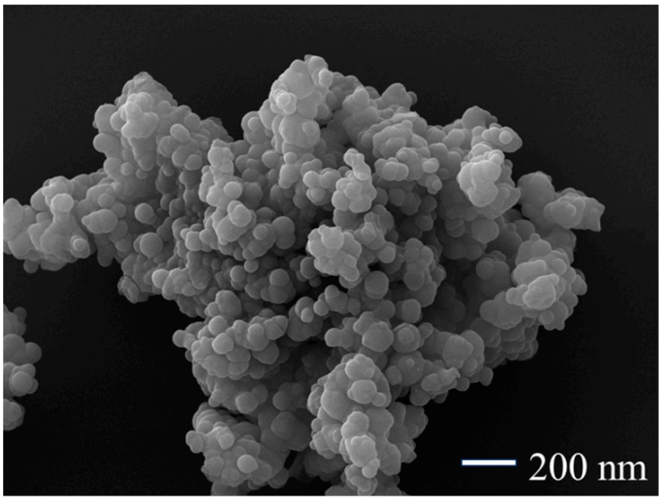

Figure S16. SEM morphology image of HB-IPOP adsorbed ReO_4^-.

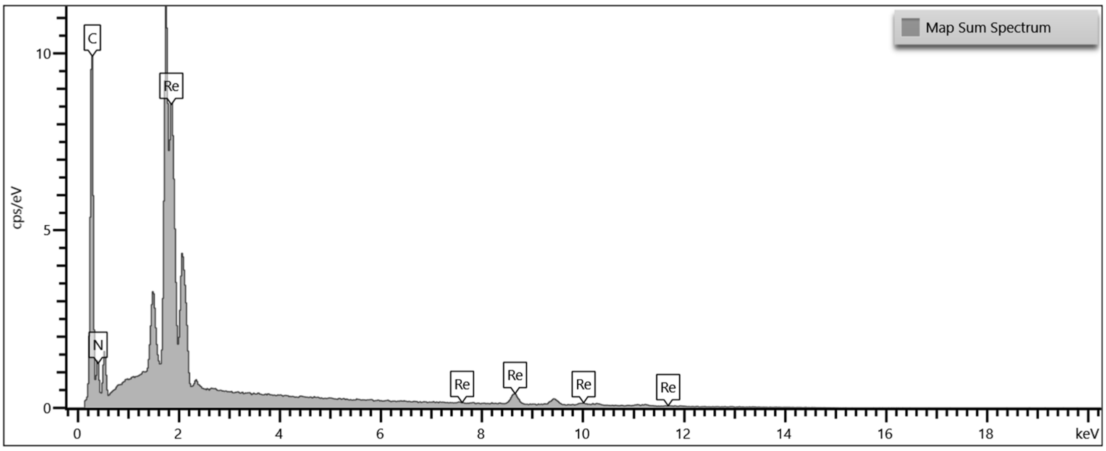

Figure S17. EDX spectra of the HB-IPOP adsorbed ReO_4^-.

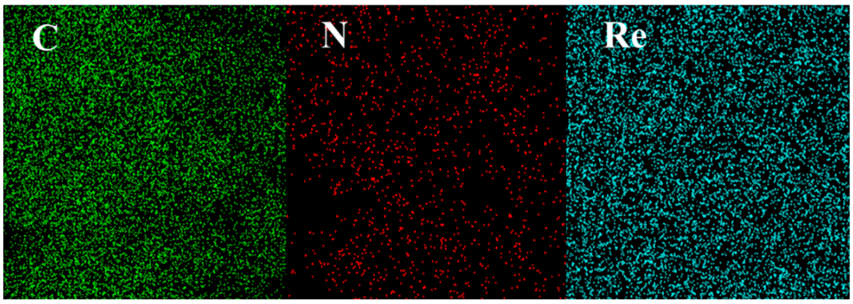

Figure S18. Elemental mapping of the HB-IPOP adsorbed ReO_4^-.

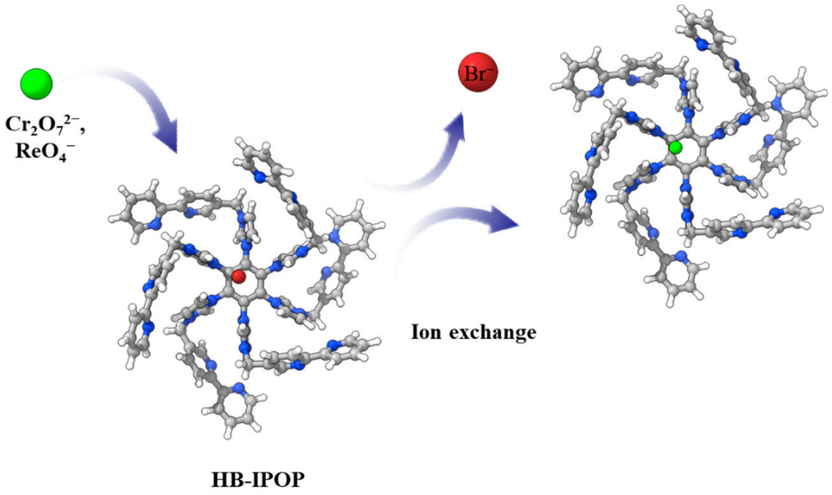

Figure 19. The adsorption process of HB-IPOP's removal of oxo-anions.

Table S1. Comparison of $Cr_2O_7^{2-}$ adsorption capacity of HB-IPOP with other adsorbents.

Materials	Adsorption capacity (mg g^{-1})	Selectivity	Reference
PVIm-6-SCD	457.8	N. D.	1
CON-LDU2	325	Cl^-, Br^-, NO_3^-, SO_4^{2-}	2
ImIP-Br	318	N. D.	3
NU-1000	76.8	Cl^-, Br^-, NO_3^-, SO_4^{2-}	4
PVIm-6-SCD	236.8	Cl^-, $H_2PO_4^-$, NO_3^-, SO_4^{2-}	1
Tp-DGCl	360.02	Cl^-, PO_4^{2-}, NO_3^-, SO_4^{2-}, MoO_4^{2-}	5
iCOF	412	Cl^-, Br^-, NO_3^-, SO_4^{2-}, BF_4^-, OAc^-, ClO_4^-	6
QUST-iPOP-1	395.96	Cl^-, Br^-, NO_3^-, SO_4^{2-}	7
FJI-C11	321	F^-, Cl^-, Br^-, NO_3^-	8
DEX-Cr	248	N. D.	9
C-NSANaph-HCP@Br	748	F^-, Cl^-, Br^-, NO_3^-, ClO_4^-, OAc^-, CO_3^{2-}, PO_4^{3-}	10
HB-IPOP	335	Cl^-, Br^-, NO_3^-	**This work**

Table S2. Comparison of ReO_4^- adsorption capacity of HB-IPOP with other adsorbents.

Materials	Adsorption capacity (mg g^{-1})	Selectivity	Reference
QUST-iPOP-1	441.6	Cl^-, Br^-, NO_3^-, SO_4^{2-}	7
iPOP-3	515.5	Cl^-, Br^-, NO_3^-, SO_4^{2-}	11
iPOP-4	350.3		
PQA-Py-Cl	849	NO_3^-, SO_4^{2-}	12
PQA-pN(Me)2Py-Cl	1127		
SLUG-21	602	NO_3^-, CO_3^{2-}	13
Compound-1	517	Cl^-, Br^-, NO_3^-, SO_4^{2-}	14
LDHs	130	N. D.	15
D318 resin	351	N. D.	16
UIO-66-NH2	159	NO_3^-, PO_3^{3-}, SO_4^{2-}	17
PAF-1-F	420	PO_3^{3-}, SO_4^{2-}	18
HB-IPOP	592	Cl^-, Br^-, NO_3^-	**This work**

References

1. Xie, Y.; Lin, J.; Liang, J.; Li, M.; Fu, Y.; Wang, H.; Tu, S.; Li, J. Hypercrosslinked mesoporous poly (ionic liquid) s with high density of ion pairs: Efficient adsorbents for Cr (VI) removal via ion-exchange. *Chem. Eng. J.* **2019**, *378*, 122107.
2. Li, Z.; Xue, H.; Ma, Y.; Zhang, Q.; Li, Y.; Xie, M.; Qi, H.; Zheng, X. Dual-functionalized fluorescent cationic organic network: Highly efficient detection and removal of dichromate from water. *ACS Appl. Mater. Interfaces* **2019**, *11*, 46197–46204.
3. Wang, Y.; Zhao, H.; Li, X.; Wang, R. A durable luminescent ionic polymer for rapid detection and efficient removal of toxic $Cr_2O_7^{2-}$. *J. Mater. Chem. A* **2016**, *4*, 12554–12560.
4. Lin, Z.; Zheng, H.; Zheng, H.; Lin, L.; Xin, Q.; Cao, R. Efficient capture and effective sensing of $Cr_2O_7^{2-}$ from water using a zirconium metal-organic framework. *Inorg. Chem.* **2017**, *56*, 14178–14188.
5. Zhuang, X.; Hao, J.; Zheng, X.; Fu, D.; Mo, P.; Jin, Y.; Chen, P.; Liu, H.; Liu, G.; Lv, W. High-performance adsorption of chromate by hydrazone-Linked guanidiniumBased ionic covalent organic frameworks: Selective ion exchange. *Sep. Purif. Technol.* **2021**, *274*, 118993.
6. Neem, M.; Mondal, M.; Pillai, R.S.; Neogi, S. Trifunctionalized ionic covalent organic framework-engineered mixed-matrix membranes for rapid and high-performance adsorptive separation of charged aqua pollutants. *ACS Appl. Eng. Mater.* **2024**, *2*, 2177–2189.
7. Jiao, S.; Deng, L.; Zhang, X.; Zhang, Y.; Liu, K.; Li, S.; Wang, L.; Ma, D. Evaluation of an ionic porous organic polymer for water remediation. *ACS Appl. Mater. Interfaces* **2021**, *13*, 39404–39413.
8. Zou, Y.; Liang, J.; He, C.; Huang, Y.; Cao, R. A mesoporous cationic metal-organic framework

with a high density of positive charge for enhanced removal of dichromate from water. *Dalt. Trans.* **2019**, *48*, 6680–6684.

9. Liu, X.; Li, Y.; Wang, C.; Ji, M. Cr (VI) removal by a new type of anion exchange resin DEX-Cr: adsorption affecting factors, isotherms, kinetics, and desorption regeneration environ. *Prog. Sus. Energy.* **2015**, *34*, 387–394.
10. Shen, X.; Ma, S.; Xia, H.; Shi, Z.; Mu, Y.; Liu, X. Cationic porous organic polymers as an excellent platform for highly efficient removal of pollutants from water. *J. Mater. Chem. A* **2018**, *6*, 20653-20658.
11. Sen, A.; Dutta, S.; Dam, G.K.; Samanta, P.; Let, S.; Sharma, S.; Let, S.; Sharma, S.; Shirolkar, M.; S.K. Ghosh, Imidazolium-functionalized chemically robust ionic porous organic polymers (iPOPs) toward toxic oxo-pollutants capture from water. *Chem. Eur. J.* **2021**, *27*, 13442−13449.
12. Sun, Q.; Zhu, L.; Aguila, B.; Thallapally, P.K.; Xu, C.; Chen, J.; Wang, S.; Rogers, D.; Ma, S. Optimizing radionuclide sequestration in anion nanotraps with record pertechnetate sorption. *Nat. Commun.* **2019**, *10*, 1646.
13. Fei, H.; Bresler, M.R.; Oliver, S.R.J. A new paradigm for anion trapping in high capacity and selectivity: Crystal-to-crystal transformation of cationic materials. *J. Am. Chem. Soc.* **2011**, *133*, 11110−11113.
14. Samanta, P.; Chandra, P.; Dutta, S.; Desai, A.V.; Ghosh, S.K. Chemically stable ionic viologen-organic network: an efficient scavenger of toxic oxo-anions from water. *Chem. Sci.* **2018**, *9*, 7874−7881.
15. Fei, H.; Han, C. S.; Robins, J. C.; Oliver, S. R. J. A cationic metal–organic solid solution based on Co(II) and Zn(II) for chromate trapping. *Chem. Mater.* **2013**, *25*, 647−652.

16. Shu, Z.; Yang, M. Adsorption of Rhenium(VII) with anion exchange resin D318. *Chinese. J. Chem. Eng.* **2010**, *18*, 372−376.

17. Banerjee, D.; Xu, W.; Nie, Z.; Johnson, L.E.V.; Cogh-lan, C.; Sushko, M.L.; Kim, D.; Schweiger, M.J.; Kruger, A.A.; Doonan, C.J.; P.K. Thallapally, Zirconium-based metal–organic framework for removal of perrhenate from water. *Inorg. Chem.* **2016**, *55*, 8241–8243.

18. Banerjee, D.; Elsaidi, S.K.; Aguila, B.; Li, B.; Kim, D.; Schweiger, M.J.; Kruger, A.A.; Doonan, C.J.; Ma, S.; Thallapally, P.K. Zirconium-based metal–organic framework for removal of perrhenate from water. *Chem. Eur. J.* **2016**, *22*, 17581−17584.
